# Enhanced antibacterial properties of polyvinyl alcohol/starch/chitosan films with NiO–CuO nanoparticles for food packaging

**DOI:** 10.1038/s41598-024-58210-8

**Published:** 2024-03-28

**Authors:** Fatemeh Momtaz, Elham Momtaz, Masoud A. Mehrgardi, Mahdieh Momtaz, Tahmineh Narimani, Farkhondeh Poursina

**Affiliations:** 1 Department of Microbiology, School of Medicine, Isfahan University of Medical Sciences, Isfahan, 81746-73461 Iran; 2 Department of Chemistry, University of Isfahan, Isfahan, 8174673441 Iran

**Keywords:** Antimicrobials, Polymer chemistry

## Abstract

Packaging is very important to maintain the quality of food and prevent the growth of microbes. Therefore, the use of food packaging with antimicrobial properties protects the food from the growth of microorganisms. In this study, antibacterial nanocomposite films of polyvinyl alcohol/starch/chitosan (PVA/ST/CS) together with nickel oxide-copper oxide nanoparticles (NiO–CuONPs) are prepared for food packaging. NiO–CuONPs were synthesized by the co-precipitation method, and structural characterization of nanoparticles (NPs) was carried out by XRD, FTIR, and SEM techniques. Composites of PVA/ST/CS, containing different percentages of NPs, were prepared by casting and characterized by FTIR and FESEM. The mechanical properties, diffusion barrier, and thermal stability were determined. The nanoparticles have a round structure with an average size of 6.7 ± 1.2 nm. The cross-section of PVA/ST/CS film is dense, uniform, and without cracks. In the mechanical tests, the addition of NPs up to 1% improved the mechanical properties (TS = 31.94 MPa), while 2% of NPs lowered TS to 14.76 MPa. The fibroblast cells toxicity and the films antibacterial activity were also examined. The films displayed stronger antibacterial effects against Gram-positive bacteria (*Staphylococcus aureus*) compared to Gram-negative bacteria (*Escherichia coli*). Furthermore, these films have no toxicity to fibroblast cells and the survival rate of these cells in contact with the films is more than 84%. Therefore, this film is recommended for food packaging due to its excellent mechanical and barrier properties, good antibacterial activity, and non-toxicity.

## Introduction

Food packaging is a barrier to protect food from spoilage and the growth of microorganisms, for this reason, choosing packaging with antimicrobial properties can be a suitable alternative for protecting food^1,2^. Plastics are the most commonly used materials for packaging due to their excellent properties, such as low cost and good mechanical, barrier, and thermal properties^3^. However, due to its long shelf life, low degradability, and negative impact on the environment, much research has been done in the field of developing biopolymers as an alternative to synthetic plastics^4^. In recent years, natural polymers, chitosan, starch, and synthetic PVA polymers have been used due to their excellent mechanical and barrier properties, ease of processing, relatively low cost, and availability^5^. Chitosan is a linear polysaccharide composed of N-acetyl-D-glucosamine and D-glucosamine units, derived from deacetylation of chitin found in the skin of crustacean (crabs and shrimps). It is the second most abundant cationic polysaccharide in the world. Chitosan has several advantages for food packaging, including antimicrobial properties, biocompatibility, degradability, nontoxicity, availability, and low cost^6^. However, despite these advantageous properties, it lacks sufficient mechanical and barrier properties. To solve this problem, it is mixed with biopolymers such as PVA. The cationic nature of chitosan causes some bonds between the amine groups of chitosan and the hydroxyl groups of PVA^7^. PVA is a water-soluble synthetic polymer obtained by the hydrolysis of polyvinyl acetate and is a suitable material for packaging^8,9^. Its good properties of PVA include biocompatibility, biodegradability, non-toxicity, odorlessness, transparency, high chemical resistance in acidic and alkaline environments, and excellent mechanical properties. PVA can be a safe material for food packaging as it is environmentally friendly and renewable^10,11^. To reduce manufacturing costs and improve mechanical properties and biodegradability, the starch biopolymer can be used. Starch is a natural polysaccharide containing the macromolecules amylose and amylopectin^12^. The starch film is inexpensive and readily available, with excellent biocompatibility and film formation, which is taken into account in food packaging^13^. However, starch films have poor mechanical properties, which limits their use as packaging^14^. To improve the mechanical resistance and barrier effect of the film, various methods have been used so far, such as combining it with other polymers. Starch and PVA form hydrogen bonds through their hydroxyl groups, improving mechanical and barrier properties. On the other hand, some interactions remain between the amine groups of chitosan and the starch hydroxyl^14^. Since the biocomposite PVA/ST/CS alone has weak antibacterial activity, metal oxide nanoparticles (NPs) are added to the polymer mixture^15,16^. Nowadays, metal oxide NPs such as nickel oxide and copper oxide with antibacterial properties are being considered for packaging. Nickel oxide and copper oxide NP have properties such as large surface area, stability, low toxicity, antibacterial activity, and availability^17–19^. Combined metal oxide NPs (NiO–CuONPs) have higher antibacterial activity against bacteria compared to single metal oxides^20^. The literature review confirms the antibacterial activity of polymers when combined with NPs^18,21–23^. To the best of our knowledge, there have been no reports on the combination of NiO–CuONPs with a PVA/ST/CS polymer blend. In this study, for the first time, a PVA/ST/CS film was synthesized together with NiO–CuONPs by casting method, and the effect of different percentages of NPs on the morphological, antibacterial, mechanical, and toxic properties of the biocomposite was investigated. We hypothesize that the inclusion of NPs in the composite enhances the antibacterial, mechanical, and barrier properties of biocomposite films, and the prepared films may be a suitable candidate for food packaging.

## Results and discussion

### Nanoparticle characteristics

**Figure 1:**
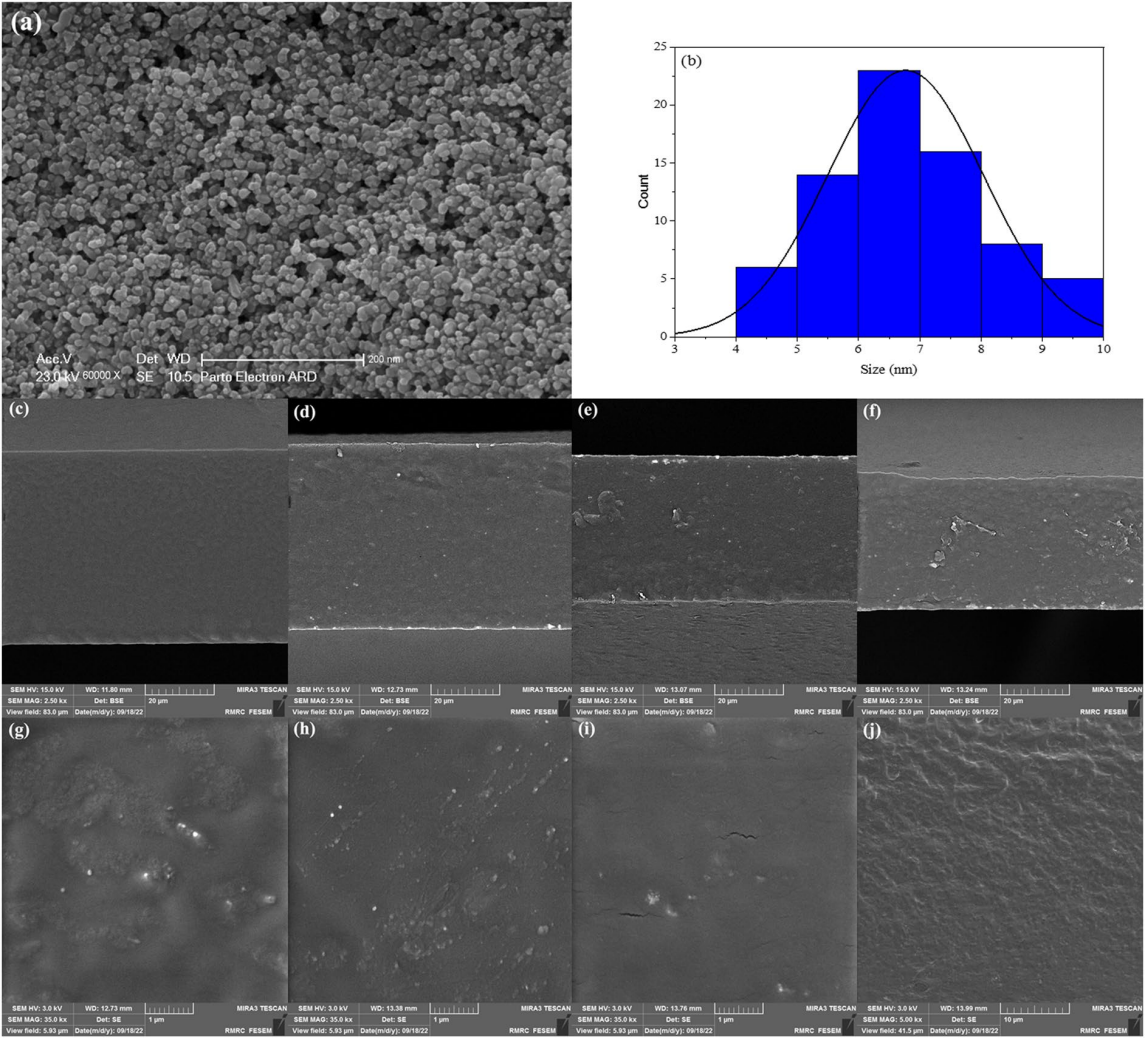
SEM image of synthesized NiO–CuONPs (**a**), size distribution histogram (**b**) and FESEM images of the cross-section surface of the bio-nanocomposite films containing 0% (**c**), 0.5% (**d**), 1% (**e**), 2% (**f**), 0.5% (**g**), 1% (**h**), 2% (**i**) and 2% of the NiO–CuONPs (**j**).

The SEM image of the synthesized NiO–CuONPs is shown in Fig. 1a. As can be seen in this figure, the spherical nanoparticles were synthesized uniformly and densely, and the histogram of particle size distribution, Fig. 1b, shows that the average size of the NPs is 6.7 ± 1.2 nm. Compared with the results reported by *Paul* and *Neogi,* the synthesized NiO–CuONPs confirm of the applied method^20^.

### Film properties

The dispersion of NPs in the bio-nanocomposite films and the morphology, including cross-sectional morphology, of these films were studied using the FESEM technique. The FESEM images of NP-free PVA/ST/CS films and PVA/ST/CS films containing 0.5%, 1%, and 2% NiO–CuONPs, respectively, are shown in Fig. 1c–f. The cross-section of the PVA/ST/CS film (Fig. 1c) is smooth and dense, without fractures or cracks, indicating strong binding interaction between the components of the polymer. The size distribution of NPs in the bio-nanocomposite films with 0.5% and 1% NPs is 38 ± 6 and 46 ± 6 nm, respectively, which are almost close to each other. In these nanocomposites, the NiO–CuONPs are uniformly dispersed throughout the composite without aggregation (Fig. 1g,h). The uniform dispersion of NPs improved the mechanical properties and antibacterial activity of the bio-nanocomposite films. The results of this study are comparable to those reported by Baghaie et al.^15^. To some extent, the addition of NPs to the polymers leads to the formation of hydrogen bonds between the polymer components and the NPs, resulting in a film with a filamentous texture. However, in this study, despite the formation of hydrogen bonds, a film with a dense and uniform texture was produced. Moreover, it was observed that with the increase of the percentage of NPs in the nanocomposite film, the size of NPs increases, and accordingly, the surface morphology of the films changes. For example, the addition of 2% NPs to a PVA/ST/CS film leads to a small accumulation of NPs (54 ± 7 nm), and some cracks are observed in the cross-sectional area of this film (Fig. 1i). The cross-section of the PVA/ST/CS/NPs 2% film (Fig. 1j) is also slightly rough. Al-Tayyar et al. and Marand et al*.* reported similar results regarding the aggregation of NPs in the polymer structure^24,25^.

### FTIR spectra

**Figure 2:**
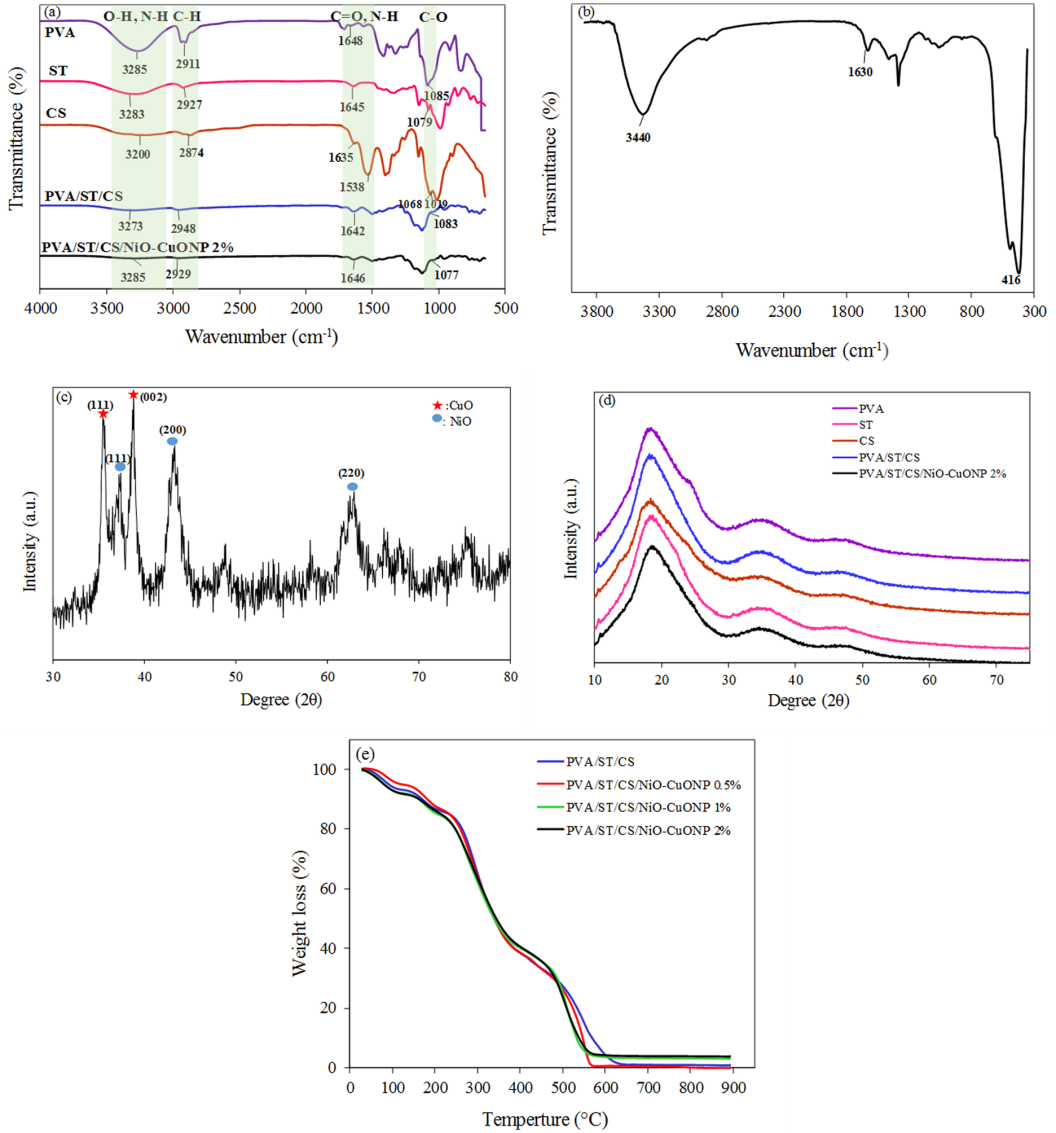
FTIR spectra of (**a**) different composite films and (**b**) NiO-CuONP; XRD patterns of (**c**) nanoparticles NiO–CuONPs, (**d**) composite films and TGA thermograms of the bio-nanocomposite films (**e**).

To decipher the structural features and functional groups of the films of PVA/ST/CS without NPs and with 2% NPs as well as NiO–CuONPs, FTIR spectra were recorded and shown in Fig. 2a,b, respectively. As shown in Fig. 2b, a broad absorption peak at 3320–3440 cm^-1^ for the NPs is attributed to the O–H stretching vibration of water molecules in the interlayers, and a weak peak at 1630 cm^-1^ is related to the H–O–H bending vibration of water molecules in the synthesized NiO–CuONPs, indicating the absorption of water molecules on the surface of the NPs^20,26^. The peaks that appeared in the range of 400–600 cm^-1^ are due to the metal oxides of the NiO–CuONPs^27^. In the FTIR analysis of bio-nanocomposites, the spectra of chitosan, starch, and PVA films are obtained by combining these polymers, e.g. PVA/ST/CS, and adding NiO–CuONPs, e.g. PVA/ST/CS/NiO–CuONPs. The spectrum of the bio-nanocomposite containing 2% NiO–CuONPs is shown in Fig. 2a as an example. From the FTIR spectrum of the chitosan film, the weak broad peak at 3300 cm^-1^, the weak narrow peak at 2874 cm^-1^, the peak at 1635 cm^-1^, the peak at 1538 cm^-1^, and the peaks at 1019 cm^-1^, 1065 cm^-1^, and 890 cm^-1^ belong to the stretching of O–H and N–H, CH, amide I, amide II (amino NH), and C–O groups, respectively. The peak at 1410 cm^-1^ is also due to the bending of the C-H group^28–32^. In the spectrum of the PVA film, the broad peak at 3285 cm^-1^ is due to the stretching of the O–H group, indicating the presence of water molecules in the structure. The vibrational peak in the 2911 cm^-1^ region is attributed to the asymmetric stretching mode of the methyl group. Also, the peaks at 1416 cm^-1^ and 1085 cm^-1^ are related to the bending vibration of the CH_2_ group and the stretching vibration of the C–O and C–H bonds, respectively^28,33,34^. From the FTIR spectrum of the starch film, it can be deduced that the weak broad peak at 3283 cm^-1^ is related to the hydroxyl group (O–H stretching) and the small peak at 2927 cm^-1^ is characteristic of C-H stretching. The peaks at 1645 cm^-1^, 1413 cm^-1^, and 1079 cm^-1^ are related to the bending of the hydroxyl group, the bending of the CH_2_ group, and the vibrational stretching of the C-O bond, respectively^35^. The recorded FTIR spectrum of the PVA/ST/CS film (Fig. 2a) shows that the broad peak in the range of 3287–3199 cm^-1^ is related to the stretching vibration of O–H and N–H, which shifts to a smaller wavenumber and indicates the reactions between the polymers during the combination process. These reactions are attributed to the new interactions between the hydroxyl groups of the chitosan and PVA chains. On the other hand, the results show that the peak at 1635 cm^-1^, C=O stretching of the amide II, in the FTIR spectrum of chitosan is transformed into 1642 cm^-1^ in the PVA/ST/CS composite, which could be due to the formation of a strong hydrogen bond between the starch functional group OH and the NH_2_ and C=O functional groups of chitosan. The absorption peak corresponding to the stretching of the O–H group is slightly shifted from 3273 cm^-1^ (PVA/ST/CS) to 3285 cm^-1^ (PVA/ST/CS/NiO–CuONPs2%). This movement to larger wavenumbers may be caused by the formation of hydrogen bonds between OH groups on the surface of the NiO–CuONPs and corresponding functional groups within the PVA/ST/CS film. Therefore, variations in the spectra belonging to hydroxyl, amine, and amide groups attached to PVA/ST/CS/NiO–CuONPs 2% bio-nanocomposites show the chemical interactions between NiO–CuONPs and the aforementioned functional groups of the polymers.

### XRD

The X-ray diffraction patterns of NiO–CuONPs and films of polyvinyl alcohol, chitosan, starch, and PVA/ST/CS with and without 2% NPs are shown in Fig. 2c,d. In Fig. 2d, the XRD pattern of the PVA film has a sharp broad peak at 2θ = 19.1° and two small peaks at 2θ = 24.1° and 2θ = 35.6° indicating the semi-crystalline structure of the polyvinyl alcohol. However, a single broad peak at 2θ = 19° in the X-ray diffraction pattern of the chitosan film indicates the amorphous structure of this polymer. In the case of the starch film, a peak appears at 2θ = 19.4°, which is characteristic of the amorphous microstructure of this file. The heating and gelatinization of the starch destroyed the semi-crystalline regions of the starch grains, resulting in an amorphous film^36–38^. The XRD pattern of the PVA/ST/CS mixture shows a broad peak at 2θ = 19.2° with greater intensity compared to the individual components, which is due to the interactions between chitosan and starch and polyvinyl alcohol. In the diffraction pattern of NiO–CuONPs, two peaks at 2θ = 38.73° and 2θ = 35.49° can be seen, respectively, corresponding to the (111) and (002) planes of the monoclinic structure of copper oxide (ICDD PDF No.00-045-0937). In addition, the observed peaks at 37.32°, 43.27°, and 62.875° correspond to the (111), (200), and (220) planes of the cubic structure of nickel oxide (ICDD PDF No.01-071-1179)^20,39^. The absence of specific peaks in the XRD pattern associated with impurities indicates the purity and crystallinity of the synthesized NPs. When 2% of the NPs are added to the polymer mixture, no characteristic peak belonging to the NPs is observed. The peak at 2θ = 19.3° with less intensity and shifted to the larger diffraction angles compared to that of the NP-free polymer, is probably due to the formation of hydrogen bonds between the components of the polymer and the NPs, which changes the structure of the films. The absence of characteristic peaks of NPs is not due to the degradation of the crystallinity of NP but to the low percentage of NPs, which is below 5% and thus below the detection limit of the diffractometer.

### Thermal analysis

The TGA curves of the PVA/ST/CS mixture and the bio-nanocomposite films (PVA/ST/CS/NPs 0.5%, PVA/ST/CS/NPs 1% & PVA/ST/CS/NPs 2%) are shown in Fig. 2e so that the thermal stability of the films can be evaluated. From these thermograms, four decomposition stages can be observed, including evaporation of water (60–110 °C), evaporation of water absorbed in the pores deep in the nanocomposites (120–175 °C), decomposition of the main chain of the polymers (240–470 °C), and decomposition of the bio-nanocomposites (500–660 °C)^40,41^. As can be seen in this figure, the addition of NiO–CuONPs to the PVA/ST/CS mixture slightly increases the thermal stability of the bio-nanocomposites. This improvement observed is primarily due to chemical interactions between the polymer chains that hinder the degradation of the polymer blend. Due to their good thermal stability, the produced bio-nanocomposite films are promising for food packaging.

### Mechanical properties

Packaging films should be able to withstand normal stresses during transportation and storage to maintain food integrity and quality. The calculated mechanical parameters such as TS and EAB for polymer films, are listed in Table 1. These parameters generally define the mechanical properties of a particular film in terms of film strength and flexibility, which are closely related to the physicochemical structure of the film. As can be seen in Table 1, increasing the chitosan percentage significantly decreases TS (*P* ˂ 0.05), while EAB does not change significantly (*P* > 0.05), since increasing the chitosan percentage leads to more network pores and less crosslinking^15^. Due to the great importance of TS for packaging, the PVA/ST/CS 1% polymer film was chosen from now on instead of the PVA/ST/CS 2% film to investigate the effects of different percentages of NPs on mechanical and antibacterial properties. Therefore, other tests related to food packaging were also performed on these samples. As Table 1 shows, the TS and EAB of the bio-nanocomposite films increase up to 1% and then decrease with an increase in the percentage of NPs. The TS and EAB of the films increase with the addition of NPs up to 1%, which could be due to the homogeneous dispersion of NPs in the polymer and the emergence of more attractive interactions between the NPs and the functional groups of the polymers^41,42^. The addition of 2% NPs to the PVA/ST/CS polymer mixture significantly decreases TS and EAB compared to the control film (*P* ˂ 0.05), which may indicate the accumulation of NPs and less hydrogen bonding between NPs and polymer functional groups^43^. Consequently, up to 1% NP in the PVA/ST/CS polymer blend improves the mechanical properties of the films, while the addition of more NP makes the films brittle.

**Table 1:** Results of WVP, MA, WS and mechanical properties of the bio-nanocomposite films.

Sample	WVP (g mm/m^2^ day kPa)	MA (%)	WS (%)	EAB (%)	TS (MPa)	Thickness (mm)
PVA/ST/CS2%	–	–	–	14.17 ± 0.3^a^	14.82 ± 0.03^a^	0.03
PVA/ST/CS1%	0.62 ± 0.05^a^	8.16 ± 0.11^a^	35.2 ± 0.96^a^	13.44 ± 0.57^a^	21.39 ± 0.8^b^	0.03
PVA/ST/CS /NiO–CuONP 0.5%	0.52 ± 0.03^b^	4.68 ± 0.64^b^	30.5 ± 1.27^b^	15.09 ± 0.25^b^	23.11 ± 1.88^b^	0.03
PVA/ST/CS /NiO-CuONP 1%	0.45 ± 0.05^c^	2.68 ± 0.58^c^	27.0 ± 1.26^c^	15.28 ± 0.97^b^	31.94 ± 1.58^c^	0.03
PVA/ST/CS /NiO–CuONP 2%	0.51 ± 0.01^b,c^	3.36 ± 0.85^c^	29.2 ± 1.35^d^	8.19 ± 0.29^c^	14.76 ± 0.49^d^	0.03

Data are expressed as mean ± standard deviation (n = 3) and different letters show significant difference at the 5% level.

### Water resistance properties

#### Water vapor permeability

The permeability of water vapor through food packaging films is a crucial factor in preventing the diffusion of evaporated water molecules to the outside or vice versa. Therefore, it is necessary to evaluate the WVP of the films. The WVP of the films without NPs and the films with different percentages of NPs (e.g., 0.5%, 1%, and 2%) is shown in Table 1. As shown in this table, the addition of NiO–CuONPs up to 1% improves the barrier properties of the films by forming a more compact structure (less empty spaces and more hydrogen bonds between the NPs and the polymer), resulting in significantly (*P* < 0.05) lower diffusion of water molecules^44–46^. However, the addition of 2% NPs to the film leads to a significant increase (*P* ˂ 0.05) in WVP. This result could be due to the non-uniform distribution of NPs in the bio-nanocomposite film, which leads to the accumulation of NPs in film^47^. Food packaging materials should have low WVP to prevent moisture exchange between food and the environment.

#### Moisture absorption

The low MA of the food packaging material is the key characteristic of these materials for suitability as food packaging. The reduction of MA helps to maintain the quality of the packaged products. In the present study, the MA values of PVA/ST/CS films were measured and listed in Table 1. The amount of MA of PVA/ST/CS film (control film) was 8.15% after 5 days. The addition of NiO–CuONPs (different percentages, e.g., 0.5%, 1%, and 2%) to the biocomposite film significantly decreased the MA in all cases compared to the control film (*P* ˂ 0.05). The addition of NPs to the film increases the mechanical properties of the film, improves its resistance to moisture, and increases its stability in a high-humidity environment. From a molecular point of view, the decrease in MA is due to the formation of hydrogen bonds between the NPs and the PVA/ST/CS mixture and the filling of voids in the polymer structure by the NPs, all of which reduce the voids and ultimately reduce the diffusion of water molecules into the film. In this way, the water resistance of the film is improved. These results are somewhat consistent with the reports in the literature^25,48^.

#### Water solubility

One of the most important properties of films or food coatings is their solubility at high/low humidity. The solubility of films in water provides information about the affinity of the polymers for water molecules. Table 1 lists the WS of PVA/ST/CS films and PVA/ST/CS films with varying percentages of NPs. According to the results presented in this table, the addition of 0.5% and 1% NPs to PVA/ST/CS film leads to a decrease in WS, which could be due to the formation of hydrogen bonds (H∙∙∙∙O interactions) between polymers and NPs and, consequently, to the hindrance of polymer chain movements. These bonds between NPs and polymer structures improve the resistance of the films in water^49^. However, the addition of 2% NPs to the film leads to an increase in WS, which could be due to the dissolution of the accumulated NiO–CuONPs on the surface of the film and a decrease in hydrogen bonding between the polymer components and the NPs^25^.

### Antimicrobial properties

Antibacterial properties play a crucial role in food packaging to bacterial growth. We evaluated the antibacterial activity of PVA/ST/CS composite films (control film) and PVA/ST/CS films containing different percentages of NPs (e.g., 0.5%, 1%, and 2%). Using the disk diffusion method, we tested these films against Gram-negative bacteria, *E. coli*, and Gram-positive bacteria, *S. aureus* which serve as food indicator bacteria. The control film shows an inhibitory effect on both bacterial strains due to the inherent antibacterial activity of chitosan. Chitosan is positively charged amino groups interact with negatively charged groups on the surface of the bacteria, leading to damage to the bacterial membrane. Thus, prevention of RNA synthesis and leakage of the cellular material leads to the destruction of the bacteria^50,51^. As Table 2 illustrates, the presence of NiO–CuONPs in the PVA/ST/CS mixture increased the antibacterial activity, which was due to the synergistic effect of NPs with chitosan^52^. The addition of NiO–CuONPs to the mixture significantly (*P* < 0.05) increased antibacterial activity in the films. The antibacterial activity of NPs is based on several mechanisms: (1) Electrostatic attraction forces between NPs and the cell wall can lead to cell wall destruction and apoptosis. (2) The binding of Ni^+2^ and Cu^+2^ ions to the negatively charged bacterial cells leads to membrane damage and leakage of cell material, ending with cell destruction. (3) The production of reactive oxygen species (ROS) induces cellular oxidative stress and dysfunction of vital macromolecules such as proteins, DNA, enzymes, and lipids, which impede cell growth or lead to death^53^. In the current study, the bio-nanocomposite films with NiO–CuONPs exhibited superior antibacterial activity against Gram-positive bacteria compared to against Gram-negative bacteria, which can be attributed to the differences in the wall structure of Gram-negative and Gram-positive bacterial cells. Gram-negative bacteria have a bilayer with a thin peptidoglycan layer and an outer membrane containing negatively charged lipopolysaccharides, thus limiting the permeability of ROS and Ni^+2^ and Cu^+2^ ions into the bacterial cell. In contrast, Gram-positive bacteria with a multilayered, less negatively charged, thick cell wall are more permeable and consequently more sensitive to NPs. The actual mechanism of interaction between NPs and ROS with bacterial cells is still under investigation^54^. Alizadeh sani et al. and Sani et al. reported similar results on the antibacterial effect of polymer film with nanoparticles against *S. aureus* and *E. coli*^46,55^. Thus, the results show that biocomposite films with NiO–CuONPs can be promising as food packaging materials, which can protect food from microbes and extend its shelf life.

**Table 2:** Antibacterial activity of bio-nanocomposite films.

Samples	Inhibitory zone (mm)	
	*S. aureus*	*E. coli*
PVA/ST/CS (Control)	9.00 ± 0.15^a^	9.00 ± 0.21^a^
PVA/ST/CS/NiO–CuONP 0.5%	10.00 ± 0.15^b^	10.00 ± 0.25^b^
PVA/ST/CS/NiO–CuONP 1%	11.50 ± 0.25^c^	10.50 ± 0.12^c^
PVA/ST/CS/NiO–CuONP 2%	13.00 ± 0.23^d^	11.00 ± 0.15^d^

Data are expressed as mean ± standard deviation (n = 3) and different letters show significant difference at the 5% level.

### Cytotoxicity evaluation

**Figure 3:**
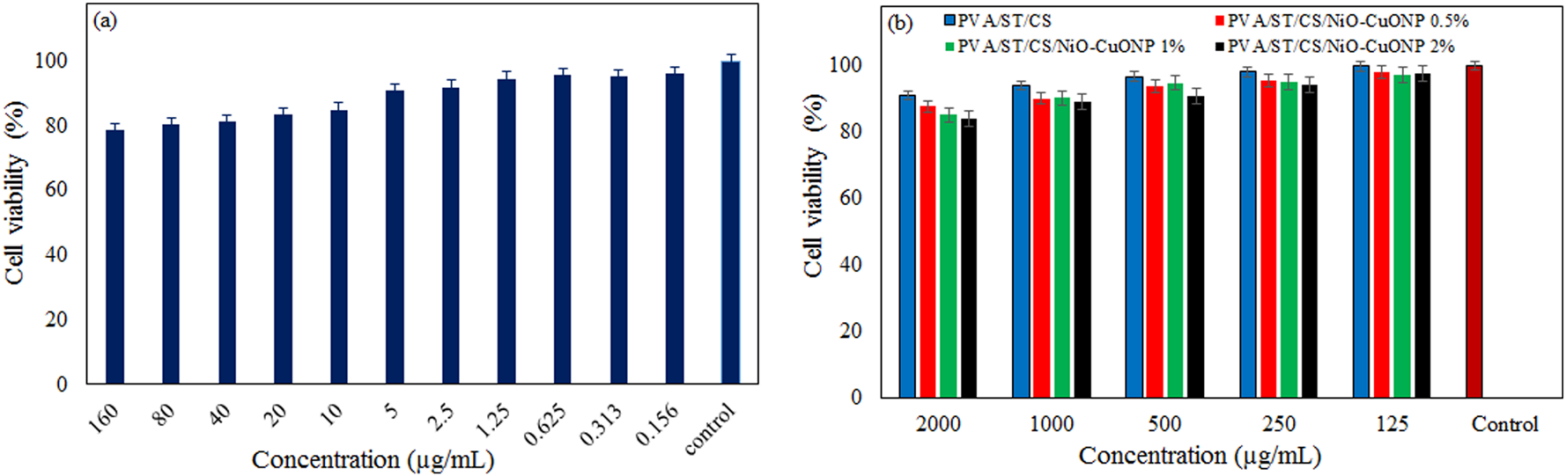
Cell viability of NIH3T3 cells in contact with NPs (**a**) and bio-nanocomposites with various percentages of NiO–CuONPs (**b**).

Food packaging polymers must be biocompatible and non-toxic as they come into contact with human skin. Therefore, the toxic effects of food packaging materials have been researched using skin cells. The toxicity effect of the nanocomposite films with NiO–CuONPs was evaluated by the cytotoxicity method on the NIH3T3 fibroblast cell line, and the percentage of cell viability was calculated. Figure 3a shows the percentage viability of NIH3T3 fibroblast cells under different concentrations of NiO–CuONPs during the cytotoxicity assay. As can be seen from this figure, after 24 h, cell viability remains at 78% for a concentration of 160 µg/mL and increases to 96% for a concentration of 0.156 µg/mL, and no toxicity is observed at low concentrations. Figure 3b illustrates the viability of cells in exposed to PVA/ST/CS films at different concentrations, (e.g. 125, 250, 500, 1000, and 2000 µg/mL). The viability of cells in contact with the NPs themselves is lower than in contact with the composite mixture containing some percentages of NPs. This result may be due to the limitations imposed on the NPs by the polymer chains so that they cannot directly contact the cells, which in turn reduces the toxicity of the films for NIH3T3 cells. These results are consistent with those published in the literature^56^. According to these results on the toxicity behavior of PVA/ST/CS/ NiO–CuONPs nanocomposite films, these films are not toxic and therefore can be used for food packaging.

## Conclusion

Packaging is an important barrier to external spoilage factors and the growth of microorganisms within the food. NiO–CuONPs were synthesized and incorporated into a PVA/ST/CS polymer film a produce a bio-nanocomposite for food packaging by the casting method. FTIR and TGA analyzes were used to determine the interaction between the nanoparticles and the polymer components as well as the good thermal stability of the films. In addition, the FESEM results show that the films with a nanoparticle content of up to 1% have a dense structure and the nanoparticles are evenly distributed throughout the composite without aggregation. Consequently, the mechanical and barrier properties of the films were enhanced by the addition of nanoparticles. In addition, the antimicrobial properties of the polymer films against Gram-positive bacteria compared to Gram-negative bacteria were enhanced by the addition of nanoparticles to the films and the viability of fibroblast cells was increased. In general, among the films prepared, the PVA/ST/CS/NiO-CuONP 1% film showed good mechanical strength, water resistance and antibacterial activity and can be recommended for food packaging.

## Methodology

### Materials

The low molecular weight chitosan (50,000–190,000 Da, degree of deacetylation (75–85%)), nickel (II) nitrate hexahydrate (Ni(NO_3_)_2_.6H_2_O), and copper (II) nitrate trihydrate (Cu(NO_3_)2)0.3H_2_O) were purchased from Sigma-Aldrich(America). Polyvinyl alcohol (98% hydrolysis degree, Mw = 22,000) and potato starch were obtained from EXIR (Austria) and HIMERDIA (India), respectively. Sodium hydroxide (NaOH), acetic acid glacial (99.9%), calcium sulfate (CaSO_4_), calcium nitrate (Ca(NO_3_)_2_), potassium sulfate (K_2_SO_4_), ethanol (96%), glycerol, dimethyl sulfoxide (DMSO) were purchased from Merck (Germany). Roswell Park Memorial Institute (RPMI 1640), Fetal bovine serum (FBS), Penicillin–Streptomycin (100x), Trypsin–EDTA (1x), 3-(4,5-dimethylthiazol-2-yl)-2,5-diphenyltetrazolium bromide (MTT) were purchased from BioIdiea Co.(Iran). The standard strains of *E. coli* (ATTC 25,922), *S. aureus* (ATTC 25,923), and mouse NIH/Swiss embryo (NIH3T3) were purchased from the Institute Pasteur Iran.

### Nanoparticles synthesis

The synthesis of NiO–CuONPs was carried out for the first time by the following method: Initially**,** 10 mL of nickel nitrate solution (0.25 M) and 10 mL of copper nitrate solution (0.25 M) were mixed in a 1:1 ratio. Subsequently, 100 mL of 0.5 M sodium hydroxide solution was added dropwise to the solution on a heating stirrer with vigorous stirring at 60 °C for 2 h. The solution’s color transitioned from blue-green to dark green. Next, the precipitate on the Whatman paper was washed four times with water and ethanol to remove unreacted substances. The obtained sediment was heated in an oven at 80 °C for 3 h to remove excess water. Afterward, the dried sediment was crushed in a porcelain mortar and the resulting powder was poured into a porcelain crucible. The sample was then fired at a temperature of 400 °C for 4 h. Finally, dark brown NiO–CuONPs were synthesized by slightly modifying the method mentioned in the literature^20^.

### Films preparation

To prepare 1% w/v and 2% w/v chitosan solutions, 1 g of chitosan was stirred in distilled water containing 1% v/v acetic acid for 1 h at room temperature. Simultaneously, 0.375 g of starch was stirred in distilled water at a temperature at 90 °C for 1 h to obtain a 1.5% w/v starch solution. Additionally, 0.5 g of PVA was stirred in distilled water at 70 °C for 1 h until a solution of 1% w/v was obtained. Next, we prepared a PVA/ST/CS 1% solution in a 4:2:4 ratio. Subsequently**,** different percentages of NiO–CuONPs (0.5%, 1%, and 2% w/w) based on dry chitosan, starch, and PVA were added to the prepared mixture. The solution was stirred at 70 °C for 1 h until a homogeneous solution was obtained. Finally, 15 mL of the mixture was poured into a polystyrene plate with a diameter of 8 cm and dried at laboratory temperature, e.g. 25 °C^57^.

### Characterization techniques

The size of NPs was determined by scanning electron microscope (SEM) using model LEO1430VP (Catlzeiss, MT, Germany). For imaging, the samples were coated with gold, and a series of images were acquired at a voltage of 15 kV. In addition, the morphology of the cross-sectional area of PVA/ST/CS films was studied using a field emission scanning electron microscope (FESEM) (model: MIRA3, TESCA, Czech). To prepare the cross sections, the films were immersed in liquid nitrogen and broken manually. The film pieces were then fixed on a holder with double-sided carbon adhesive and then covered with platinum. Imaging was performed with an accelerating voltage of 5 kV. Fourier transform infrared spectroscopy (FT-IR) with a Perkin Elmer spectrometer in the wavenumber range 400–4000 cm^-1^ was used to determine the spectrum of the film's functional groups. X-ray diffraction patterns were recorded using a diffractometer (Rigaku Ultima Iv, Japan) with a copper beam at 40 kV and a nickel filter, in the temperature range of 10–80 °C. Thermal analysis of the composite films was performed using the thermogravimetric analyzer (STA6000, Perkin Elmer, Waltham, Massachusetts, USA). In this analysis, approximately 2.5 mg of the film was subjected to heating rate of 10 °C/min within the temperature range of 30–900 °C under a nitrogen atmosphere.

### Mechanical properties

The biocomposites of PVA/ST/CS with different percentages of chitosan and NPs were subjected to tensile loading to evaluate their mechanical properties. Using the instrument STM-150 device, the mechanical properties of the bio-nanocomposite films, such as tensile strength (TS) and elongation at break (EAB), were investigated three times for each sample according to the ASTM standard, and the average values were reported along with the standard deviations. The films were cut with dimensions of 10 × 60 mm^2^ and stretched at a rate of 1 mm/min with a length gauge of 40 mm^40^.

### Water resistance properties

In this study, the water resistance properties of the prepared films were investigated in terms of water vapor permeability (WVP), moisture absorption (MA), and water solubility (WS). ensure test repeatability, all experiments were repeated three times and the averaged data along with their standard deviations were reported accordingly.

#### Water vapor permeability (WVP)

The WVP test was performed according to the ASTM E96-05 standard^49^. For this purpose, glass vials with an opening diameter of 2 cm and a height of 4.5 cm were used. In each vial, 3 g of anhydrous calcium sulfate was poured at a relative humidity of 0%. First, the samples were cut round, slightly larger than the opening diameter of the vial, and placed on the open side of the vial. Then, the vials were weighed along with the entire contents. The vials were stored in a desiccator with saturated potassium sulfate with a relative humidity of 97%. A small amount of solid potassium sulfate formed at the bottom of the desiccator, confirming a saturated solution. The desiccator was placed in an incubator at a temperature of 25 °C. The weight of the vials was measured every 24 h for 7 days, the evolution of weight over time was recorded, and linear regression slopes were calculated (weight versus time). The water vapor transmission rate (WVTR) was derived by dividing the slope of each vial (gr/day) by the transmission area (m^2^). Finally, the WVP (g mm m^-2^ day^-1^ kPa^-1^) of the films was calculated using the following formula:

1 $${\text{WVP}}=\frac{WVTR \times X}{P ({R}_{1}-{R}_{2})}$$

equation where X is the film thickness (mm), P introduces the saturated water vapor pressure at 25 °C (3.169 kpa), and finally, R_1_ and R_2_ represent the RH in the desiccator (RH = 97%) and RH in the vial (RH = 0%), respectively.

#### Moisture absorption (MA)

The MA method applied in this study is based on the method introduced by Amjadi et al.^48^. Composite films with dimensions of 20 × 20 mm^2^ were cut and stored in a desiccator with calcium sulfate under 0% relative humidity for 24 h. Next, the films were weighed and transferred to another desiccator with a saturated calcium nitrate solution at a relative humidity of 55% and a temperature of 25 ˚C. Over 7 days, the films were weighed every 24 until they reached a constant weight. Finally, MA was calculated according to the following formula:

2 $$\mathrm{MA }\left(\mathrm{\%}\right)=\frac{{W}_{t}-{W}_{0}}{{W}_{0}}\times 100$$

where w_t_ and w_0_ remain the final and initial weights, respectively.

#### Water solubility (WS)

Following the method introduced by *Peighambardoust *et al*.*, WS was measured for composite films^49^. A series of film samples with a size of 2 × 2 cm^2^ was prepared. Then the samples were placed in an oven (105 °C) for 1 h and simultaneously each sample was weighed to reach a constant weight (m_0_). Then, these films were immersed in 50 mL of distilled water for 24 h. After 24 h, the remaining pieces of the films were removed from the water and placed in a 105 °C hot oven to dry and reach a constant weight (m_1_). WS (%) was calculated according to the following formula:

3 $$\mathrm{WS }(\mathrm{\%})=\frac{{m}_{0}-{m}_{1}}{{m}_{0}}\times 100$$

### Antimicrobial test

The antibacterial activity of the bio-nanocomposite films was investigated by the disk diffusion method against standard strains of *E. coli* (ATCC25922) and *S. aureus* (ATCC25923). Tryptic soy broth medium was used as the nutrient source for bacterial cultivation. The bacterial suspension was prepared in physiological serum at a concentration of 10^8^ CFU/mL. Then 100 µl of it was transferred to Mueller Hinton Agar (MHA) medium and cultured using a sterile swap. Films were then sterilized in the form of 5 mm diameter disks for 15 min under ultraviolet radiation. Disking was performed on the MHA medium, and the plate was incubated at 37 ˚C for 24 h. In the final stage, the films was measured the diameter of the zone inhibiting bacterial growth around^58^.

### Cytotoxicity evaluation

Toxicity testing of NiO–CuONPs and PVA/ST/CS films with different percentages of NiO–CuONPs was performed according to the ISO10993-5 standard^59^. NIH3T3 mouse fibroblast cells were used to study the toxicity of the films. First, the cells were cultured in cell flasks in RPMI1640 medium with 10% FBS and 1% antibiotics (penicillin and streptomycins 100X), then the flasks were placed in an incubator containing 5% CO_2_ and 95% humidity for 24 h at 37 °C. Then 100 µl of cells, approximately 10^4^ cells, were added to each well of a 96-well plate and incubated for 24 h. 4 mg of the films were sterilized with UV radiation for 30 min. Then the samples were dissolved in DMSO and five dilutions ranging from 2000 to 125 µg/mL of it were prepared, e.g., 2000, 1000, 500, 250, and 125 µg/mL. Then 100 μl of each dilution was added to each well and this was repeated three times. It is noteworthy that the control wells contained untreated cells at all stages of this assay. On the other hand, a nanoparticle solution with a concentration of 1 mg/mL was prepared, and different dilutions ranging from 160 to 0.156 µg/mL were made. Three replicates of each dilution, consisting of 100 µl, were added to the wells. The plates were incubated for 24 h at 37 °C, 5% CO_2_, and 95% humidity. After 24 h, the samples were replaced with 100 μL MTT and incubated for 2 h. Finally, the MTT plate was removed, and to dissolve the formazan crystals, 100 μL DMSO was added to the wells. The plate was incubated for 15 min, and the absorbance was measured using a BIORAD model 680 microplate reader at a wavelength of 570 nm. In this study, the percent viability of the cells was evaluated as follows:

4 $$\mathrm{Cell \,viability }(\mathrm{\%})=\frac{{A}_{Sample}}{{A}_{Control}}\times 100$$

### Statistical analysis

All data reported in this study were statistically analyzed by Duncan's multiple ranges and ANOVA tests in the SPSS software (version 23). These data were expressed as mean ± standard deviation (SD), and statistically significant results were defined by a value of *P* < 0.05.

## Data Availability

The datasets generated during and/ or analyzed during the current study are available from the corresponding author (F. Poursina) on reasonable request.
